# Research on a Rice Counting Algorithm Based on an Improved MCNN and a Density Map

**DOI:** 10.3390/e23060721

**Published:** 2021-06-05

**Authors:** Ao Feng, Hongxiang Li, Zixi Liu, Yuanjiang Luo, Haibo Pu, Bin Lin, Tao Liu

**Affiliations:** College of Information Engineering, Sichuan Agricultural University, Ya’an 625000, China; fengao@stu.sicau.edu.cn (A.F.); lhx@stu.sicau.edu.cn (H.L.); liuzixi0617@gmail.com (Z.L.); 201902233@stu.sicau.edu.cn (Y.L.); 201902098@stu.sicau.edu.cn (B.L.); liutao@sicau.edu.cn (T.L.)

**Keywords:** rice, thousand grain weight, density map, multi-column convolutional neural network, advanced priori

## Abstract

The thousand grain weight is an index of size, fullness and quality in crop seed detection and is an important basis for field yield prediction. To detect the thousand grain weight of rice requires the accurate counting of rice. We collected a total of 5670 images of three different types of rice seeds with different qualities to construct a model. Considering the different shapes of different types of rice, this study used an adaptive Gaussian kernel to convolve with the rice coordinate function to obtain a more accurate density map, which was used as an important basis for determining the results of subsequent experiments. A Multi-Column Convolutional Neural Network was used to extract the features of different sizes of rice, and the features were fused by the fusion network to learn the mapping relationship from the original map features to the density map features. An advanced prior step was added to the original algorithm to estimate the density level of the image, which weakened the effect of the rice adhesion condition on the counting results. Extensive comparison experiments show that the proposed method is more accurate than the original MCNN algorithm.

## 1. Introduction

China’s annual rice exports and imports are among the highest in the world. From 2019 to 2020, among the world rice importers, China ranked first in rice imports but fourth in rice exports [1], and the reason for this is the low yield of high-quality rice in China. With the continuous improvement of people’s quality of life, people’s requirements for rice quality have become increasingly stringent [2], and how to accurately estimate rice quality is a key research topic for scholars. Studies have shown that the quality of different types of rice varies according to their shapes [3]. Therefore, it is difficult to accurately determine the quality of rice from its shape. In addition to the appearance factor, thousand grain weight is also one of the indicators to judge the quality of the crop [4]. Thousand grain weight is measured in grams and represents the weight of 1000 g of the crop. Thousand grain weight is the main criterion for predicting grain production capacity, and it is also an important index for judging the size, fullness, and quality of crop seeds, so the thousand grain weight of rice reflects its quality to some extent [5]. However, to detect the thousand grain weight of rice, it is necessary to count the rice accurately.

Previously, the counting stage in the process of measuring thousand seed weight was based on manual counting, which was not only time-consuming and labor-intensive, but also had a high degree of error. Based on this, a series of instrumental devices for direct seed counting were developed, which, unlike purely algorithmic studies, have a very high counting accuracy with the help of hardware devices. Researchers have designed a fast detection method that applies machine learning to maize counting, solving the problem of laborious and time-consuming errors [6]. With the passage of time and with scientific and technological developments, various well-established image processing software has emerged to meet the needs of various disciplines. As early as 2002, image processing systems were used to accurately calculate the number of colonies [7].

Traditional counting methods utilize Machine Learning (ML), which generates sliding windows for target detection [8]. However, the concept of deep learning introduced by Geoffrey Hinton at the beginning of the 21st century revolutionized the traditional approach to machine learning [9]. Deep Learning (DL), a newly introduced area of machine learning, is hoped to be able to move closer to Artificial Intelligence (AI) and solve complex pattern recognition problems by imitating human visual and auditory senses as well as thinking processes [10].

Deep learning methods are more often applied in the field of Computer Vision (CV) than traditional computer learning methods [11]. Computer vision has been demonstrated vastly and is playing an active role in various fields. Some researchers have used image processing methods to solve the problems in manual counting. Early tumor detection in red blood cells using the digital image processing method solved the problems of expensive instrumental methods and time-consuming manual detection methods, and its recognition rate could reach 95.0–97.0% [12]. JAR Dan et al. automatically detected and identified greenhouse pests by building a cascade-based deep learning classification method, and the final accuracy rate reached 90–92% [13]. Tetila et al. evaluated convolutional neural networks with three different training strategies in order to achieve fast and accurate automatic identification and detection counts for soybean pests and achieved good results [14]. Some researchers have also designed a two-column convolutional neural network of POD images to count soybean seeds with a high recognition rate [15]. Internationally, there are also studies on quality detection using counting methods. Huang et al. counted rice by image processing and solved the problem of unsatisfactory overlapping segmentation of rice images [16].

The counting method is not only used in crop quality detection, but also can count other objects by a density map. In order to obtain an accurate density map, many scholars have studied it. In the 2017 paper entitled “Generating [a] high-quality crowd density map”, using top international conference ICCV’s CNNs through a context pyramid, the author fully considers the global density and local density information of the crowd in an image and strengthens the constraints on the density map by extracting the global and local semantic information of the image, so that the network can adaptively learn the characteristics of the corresponding density level for any image [17]. In the article “Iterative crowd counting”, included in the ECCV in 2018, the author creates a low-resolution density map, optimizes it to obtain a high-resolution density map, and inputs it into the network structure with two CNN branches for feature extraction [18].

The main contributions of this paper are as follows: we (1) produced a rice count dataset, (2) designed an adaptive Gaussian kernel function to transform the density map for different shapes of rice to enhance the migration ability of the model, (3) fused the high-level prior as an auxiliary training into the MCNN network to increase the accuracy of the results, and (4) used a 1 × 1 convolutional kernel at the end of the network to solve the problem of varying input image sizes.

This paper is structured as follows: Section 2 reviews the related methods. Section 3 details the algorithms and models used in this paper, and proposes to fuse the high-level prior to the MCNN network to improve the accuracy of counting overlapping sticky rice. Section 4 focuses on the experimental part, including a representation of the dataset, a comparison of model performance, and an analysis of the experimental results of the three rice species. In the last section, the paper is summarized, and future directions of work are proposed.

## 2. Related Work

### 2.1. Adaptive Gaussian Kernel

The adaptive Gaussian kernel used in this paper is used to estimate the size of rice in an image and to convert the original image into a density map. The Gaussian function that is often used for image processing is a two-dimensional Gaussian function with a normal distribution of the form:

(1) $$G\left(x,y\right)=\frac{1}{2\pi \sigma^{2}}e^{\frac{-(x^{2}+y^{2})}{2\sigma^{2}}}.$$

The function usually has fixed parameters when acquiring the density map, and since the image with a circular box blurred for the convolution will have a more accurate out-of-focus imaging effect, the closer the experimental object is to the circle, the better [19]. However, the size of the rice used in this experiment varies, and the shape of the rice is also far from circular. Based on the above problems, the adaptive Gaussian kernel will be used to make the density map in this paper. An adaptive Gaussian kernel is a fusion of the kernel adaptive filtering algorithm and the Gaussian kernel function, which can change the value of the Gaussian function adaptively according to the actual shape of the object. In 2004, Engel and other researchers were the first to apply the kernel method to an adaptive filter to create a kernel adaptive RLS filter [20]. The kernel adaptive filter (KAF) uses a linear adaptive filter to process nonlinear signals by fixing a mapping that takes a linear data input and maps it to a higher dimensional feature space. Using the inner product of the input data represents the most common iterative update process of the linear adaptive lateral filter weight vector. Subsequently, using a nonlinear mapping, the inner product can be mapped into a repeatable Hilbert space that holds a kernel called Mercer, which has excellent continuous, positive, and changeable properties. Based on these properties, the output of the filter after mapping to the RKHS space can be obtained directly without computing the update of the weight vector, and the kernel function mapped to the high-dimensional space can be extended to the form of eigenvalues and eigenvectors by the Mercer kernel, thus enabling the combination of the kernel method with adaptive filtering.

### 2.2. Spatial Pyramid Pooling (SPP)

In the commonly used Convolutional Neural Network architecture, a fully connected (FC) layer is added at the end of the last convolutional layer. Since the input features of the fully connected layer are decided at the beginning, a fixed-input operation is usually performed on the input image, and the input size is decided (fixed-size). However, this usually increases the workload, and if the fixed-size operation is performed on the original image, it will cause the aspect ratio of the image to change, which will cause the image pixels to change, resulting in distortion of the source image and a loss of feature information. However, SPP (Spatial Pyramid Pooling) can solve the above problems [21].

**Figure 1:**
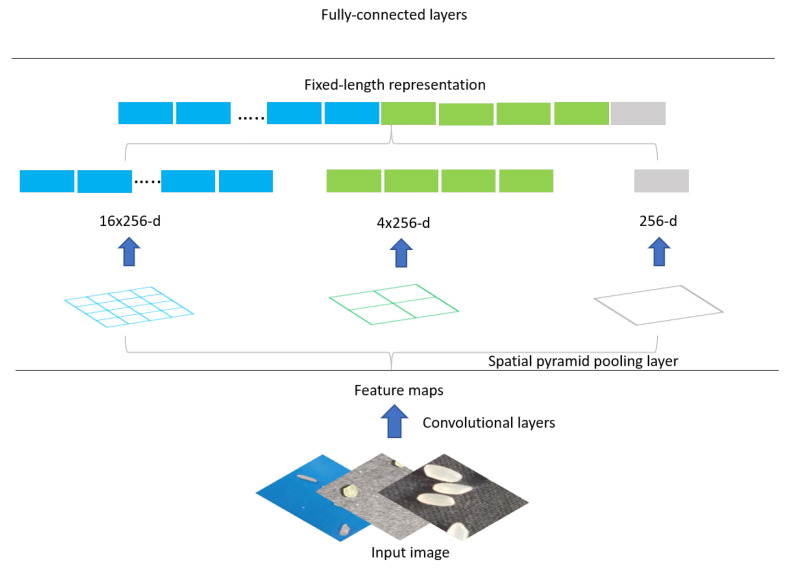
SPSS’s basic structure.

As shown in Figure 1, the Spatial Pyramid Pooling (SPP) layer structure is capable of directly accepting raw images of arbitrary size and generating fixed size outputs based on these arbitrary size images. The Spatial Pyramid layer takes the final output of the convolutional layer, a random-sized feature dimensional map, and divides it into blocks of 1, 4, and 16 sizes [22,23]. Max pooling is performed on these blocks so that a fixed number of features can be obtained after pooling, and all these features are stitched together to obtain a fixed dimensional output to satisfy the condition of fixed input dimensionality required by the fully connected layer.

### 2.3. Advanced Priori

There are three states of experience: transcendental, a priori, and a posteriori [24]. A posteriori is what we know about a thing after it is experienced. The transcendental is something beyond experience, something that ordinary people cannot experience together and thus cannot form a universal experience. A priori is known before experience, as in logic or common sense after experience. A priori is also possible in computers. The advanced prior, also called the high-level prior, in this paper is first divided into different labeled groups based on the amount of rice in the graph. Using labels, the high-level prior can roughly estimate the amount of rice in the entire photo independent of scale variation, thus allowing the network to learn more discriminative global features.

## 3. Methods and Materials

### 3.1. Experimental Dataset and Processing

**Figure 2:**
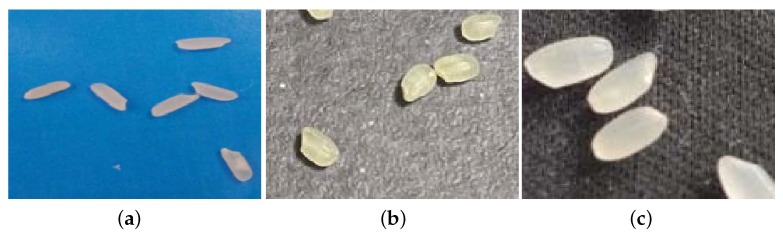
The appearance of the three types of rice. (**a**) shows indica rice; the shape is elongated, the color is creamy white, it has a transparent texture, and some of the rice will also show a white opaque texture. In (**b**,**c**), some of the rice is japonica rice; in (**b**), the rice is yellowish, has a shorter and broader shape, and is inclined toward a short oval shape; in (**c**), the rice is creamy white and has a long oval shape, a shape between (**a**,**b**).

In order to ensure the authenticity and validity of the experiments, the counting algorithm and its improvement algorithm in this paper use all the datasets we collected from rice photos. In the basic experiment stage, 5670 labeled photos of three types of rice were collected, and the complete rice centers in the photos were labeled with coordinates for the training set to learn density map features. The dataset was divided into three parts according to the species, and the specific shapes of the three types of rice are shown in Figure 2.

When collecting the dataset, in order to make this experimental algorithm practical, the diversity of the data and the robustness of the training model were increased. The photos were not taken from a single angle. There were three angles: a far top view, a near top view, and an oblique top view. In addition, the entire dataset was enhanced by dividing photos into 10 smaller photos, each of which is one-fourth of the original image size, to enrich the data to enhance the robustness of the model.

### 3.2. Density Map Generation

Since the MCNN needs to be trained to estimate the rice density map from the input image, the quality of the density map given in the training data largely determines the performance of this method. We take the following approach to convert the real image of labeled rice into a rice density map. We can suppose rice exists at the photo pixel $x_{i}$, which is represented in this paper as a function δ ($x-x_{i}$) about δ, where x represents the coordinates, and $x_{i}$ represents the actual coordinates labeled in this paper. Thus, a labeled image possessing *N* grains of rice can be expressed as the following discrete function:

(2) $$H\left(x\right)=\sum_{i=1}^{N}\delta \left(x-x_{i}\right).$$

The density function is a continuous function, so it is necessary to convert Equation (2) to a continuous function. In this paper, the Gaussian kernel $G_{\sigma }$ is used to convolve the rice discrete function [23]. It is expressed as follows:

(3) $$F\left(x\right)=H\left(x\right)*G_{\sigma }\left(x\right)$$

However, such a density function is valid only if the rice in the image plane are mutually independent samples, that is, no occlusion and no overlap. However, such a prerequisite hardly exists in realistic situations. Due to the different camera angles of the dataset, it is likely to produce perspective deformation, especially under the oblique top view. Additionally, rice itself has different shapes and sizes and occupies different areas, so the density map generated by using a fixed Gaussian kernel is not accurate. Therefore, to accurately estimate the density of rice, it is necessary to consider the effects of perspective deformation and the different shapes of rice. It has been found that, in scenes with high density, the object size is related to the distance from the center of the object [25]. In this paper, for scenes with a high rice density, the Gaussian kernel parameters are determined adaptively based on the average distance of each rice grain from its neighbors.

For each rice grain in a given image, the distances of its k rice nearest neighbors are denoted as $d_{1}^{i}$, $d_{2}^{i}$, ..., $d_{m}^{i}$ in this paper. Thus, the expression of the average distance $\overset{´}{d^{1}}$ is as follows:

(4) $$\overset{´}{d^{1}}=\frac{1}{m}\sum_{i=1}^{m}d_{j}^{i},$$

where *i* is the selected rice, and *m* is the amount of its neighboring rice. In order to estimate the density of rice around pixel $x_{i}$, in this paper, the rice dispersion function δ ($x-x_{i}$) and the distance-dependent Gaussian kernel function are convolved. More precisely, the density *F* should be expressed as follows:

(5) $$F\left(x\right)=\sum_{i=1}^{N}\delta \left(x-x_{i}\right)*G_{\sigma 1}\left(x\right),\sigma_{i}=\beta \overset{´}{d^{l}}$$

**Figure 3:**
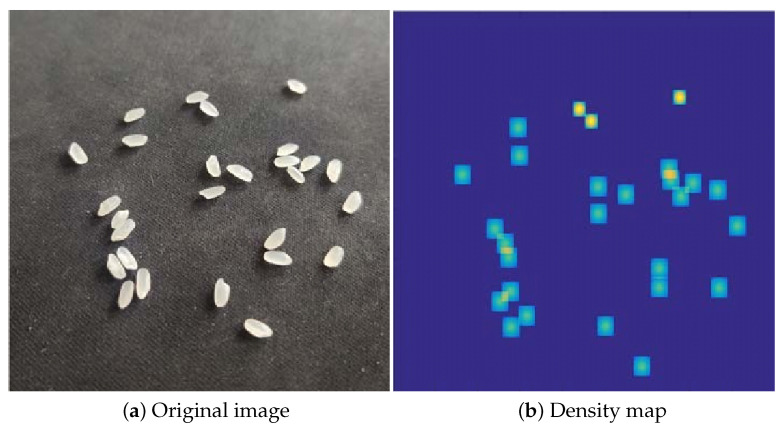
Comparison of the original image and the density map generated from the original image.

When $\overset{´}{d^{1}}$ is a certain $\sigma_{i}$, it is determined by the β parameter, which determines the Gaussian kernel function. Equations (3) and (4) represent the adaptive Gaussian kernel that has adapted to the labeled data points around the labeled data points. In this experiment, the best density map result is found when β is 0.3, as shown in Figure 3.

### 3.3. Multi-Column Convolutional Neural Network

**Figure 4:**
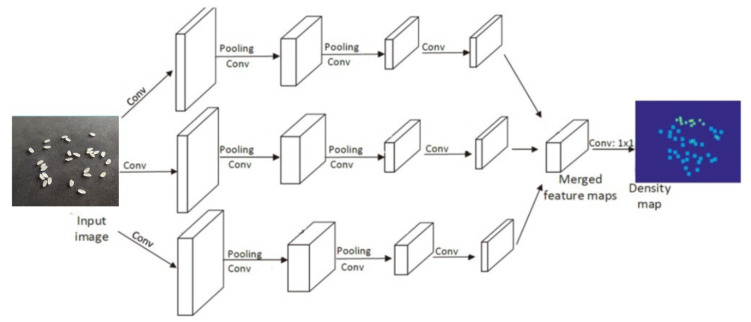
MCNN base structure, containing three parallel CNNs with filters with different sizes of local receptive fields.

The Multi-Column Convolutional Neural Network (MCNN) is a convolutional neural network model proposed on the basis of Multi-Column Deep Neural Networks (MDNNs), and we will use it to learn the target density map [26]. Due to the different shapes and sizes of the experimental rice itself and the image perspective distortion, the images contain rice of different sizes, so it is difficult for filters with receptive fields of the same size to capture the rice density features at different scales. It is more natural to use filters with local receivers of different sizes to learn the mapping from the original pixels to the density map. In the MCNN used in this paper, for each column, filters of different sizes are used to model the density maps corresponding to different scales of rice. For example, filters with larger receptive fields are more useful for modeling density maps corresponding to larger rice. The MCNN structure used in this paper is shown in Figure 4.

To reduce the computation time, we use the same Conv-Pooling-Conv-Pooling structure for all columns, which contains four convolutional processes, but the size and number of filters vary. The three side-by-side CNNs extract large, medium, and small features from top to bottom, which are called the L, M, and S rows, respectively. The first Convolution Layer in Row L uses a 9 × 9 filter with 16 channels; the second Convolution Layer is 7 × 7 with 32 channels; the third Convolution Layer is also 7 × 7 with 16 channels, and the last Convolution Layer is also 7 × 7. The first Convolution Layer in Row M uses a 7 × 7 filter with 20 channels. The first Convolution Layer in Row S uses a 5 × 5 filter with a channel of 24. The last three revolution layers use 3 × 3 filter with channels of 48, 24 and 12 respectively. All three CNNs use maximum pooling in the pooling process and use 2 × 2 size regions with rectified linear units (ReLUs) as the activation function because of its good performance for CNNs. To reduce the computational complexity (the number of parameters to be optimized), a smaller number of filters is used in this paper for CNNs with larger filters [27]. Experimentally, the feature maps of all convolutional neural network outputs are overlapped and mapped onto the density map. To map the feature maps to the density map, a filter of size 1 × 1 is used in this paper. It is important to note that traditional CNNs usually perform a step of image preprocessing, that is, images of different sizes are planned to the same size by stretching or cropping [28]. In this paper, the original size of the input image is chosen because resizing the image to the same size would introduce, in the density map, additional distortion [29]. In addition to the fact that the filters in the CNNs in this paper have different sizes, the remaining difference between the MCNN used in this paper and the normal MDNN is that the MCNN weights the outputs of the CNNs with different columns with the network learnable weights. By contrast, in the previously proposed MDNNs, the outputs are simply averaged.

### 3.4. Improved Algorithm

Although the rice counting algorithm based on the MCNN and density map can successfully count rice, its generated density map has a low resolution and tends to lose more detailed features due to the presence of the maximum pooling layer. Considering these problems, this paper will add advanced prior steps to the original algorithm MCNN and density map. In the improved algorithm, MCNN estimates the density and uses a single column of convolutional neural network to extract a high-level prior on the whole photo, and the prior part accepts the previously obtained feature map as an input. This part covers four Convolution Layers, and after each Convolution Layer is over, PReLU is used to perform activation operations on the neurons. At the beginning of the network, two Convolution Layers end with a Max Pooling operation of step size 2. At the end of the network, it is appropriate to use three fully connected layers, using the same activation function as before. In this paper, in order to enable the preprocessing operation to be performed even for images of different sizes, a spatial pyramid pooling structure is used, allowing this structure to chunk the feature maps generated by the convolution layers, producing a determined number of outputs that can be provided to the fully connected layers. Cross Entropy Loss is used as the loss function for the high-level prior part, and the approximate number of objects in the image can be estimated. The high-level prior enables the network to learn globally relevant discriminative features, which is beneficial for image object density estimation with highly variable appearance.

**Figure 5:**
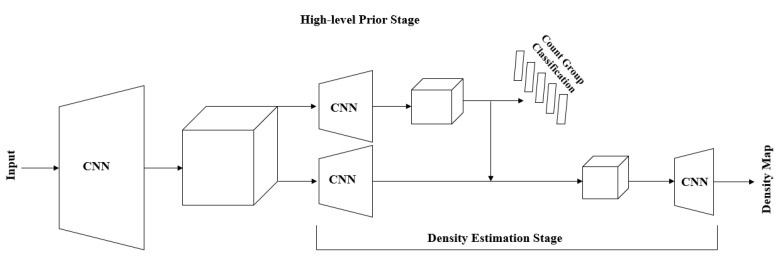
Network structure of the improved algorithm.

The advanced priori and density map estimation are two side-by-side subtasks in the improved algorithm, and the network structure of the improved algorithm is shown in Figure 5.

The upper part of the improved algorithm network structure is the network structure of the high-level prior, and the lower part also uses the MCNN to learn the feature mapping relationship from the original graph to the density graph. Unlike the original algorithm, when learning the feature mapping relationship from the original map to the density map, the first part of the high-level prior will incorporate the learned results into the mapping relationship learning, that is, the subsequent MCNN training of the improved algorithm will use the results as the benchmark. At the same time, using small-step convolutional layers, the output of the previous layer will employ up-sampling, so that the low-resolution problem caused by the maximum pooling layer can be compensated.

In the improved algorithm, a classifier is learned, and it is used to classify the amount of rice in each graph, determine the range of the amount of rice in each graph, and improve the accuracy, while the classifier also performs the task of merging high-level priors into the network. The MCNN network that generates the density maps is still composed of three side-by-side CNNs, including four Convolution Layers, each of which ends with a PReLU as the activation function. The initial two CNNs end with a Max Pooling layer with a step size of 2. The first Convolution Layer is 7 × 7 with 20 channels, the second is 5 × 5 with 40 channels, the third is 5 × 5 with 20 channels, and the fourth is 5 × 5 with 10 channels. The output of this network is combined with the output of the high-level prior through two Convolution Layers and two small-step convolutional layers. The first two Convolution Layers are 3 × 3, 24 channels and 32 channels, and the small-step convolution layers are 16 channels and 18 channels. These small-step convolution layers, in addition to integrating the prior, can also boost the feature map to the original input size, thereby recovering the detail previously lost in the maximum pooling layers to recover the details lost in the previous maximum pooling layer. The use of these layers increases the upsampling rate of the MCNN output by a factor of 4, allowing us to regress on the full-resolution density map. Standard Euclidean loss is used as a loss layer.

The cross-entropy loss function for the advanced prior part is shown below:

(6) $$L_{c}=\frac{-1}{N}\sum_{i=1}^{N}\sum_{j=1}^{M}\left[\left(y^{i}=j\right)F_{c}\left(X_{i},\theta \right)\right],$$

where *N* denotes the number of training examples, θ is again a set of MCNN deriving network parameters, $X_{i}$ is the *i*-th training example and denotes the output classification, $F_{c}\left(X_{i},\theta \right)$ is the truth classification, and $y^{i}$ and *M* is the number of categories. The density estimation loss function is shown as follows:

(7) $$L_{d}=\frac{1}{N}\sum_{i=1}^{N}\left|\right|F_{d}\left(X_{i},C_{i},\theta \right)-D_{i}{\left|\right|}_{2},$$

where $F_{d}$ is the estimated density map, $D_{i}$ is the true density map, and $C_{i}$ is the feature map derived from the last convolution layer of the advanced prior stage. The total loss function is shown as follows:

(8) $$L=\lambda L_{2}+L_{d}.$$

This loss function differs from traditional multitask learning because the loss term in the last stage depends only on the output of the previous stage.

## 4. Results and Discussion

In order to verify the effectiveness of the improved model in this paper, we first experimented the model on rice data coming from three different categories and selected the prediction results as a comparison. We used ACC, MSE, and MAE as the main evaluation indexes. The ACC is the accuracy rate, which reflects the proportion of the number of correct predicted rice categories to the total. MAE and MSE denote the absolute mean error and mean squared error of the best models of rice in the training phase for Categories A, B, and C, respectively. They are calculated as follows:

(9) $$ACC=\frac{T_{P}+T_{N}}{T_{P}+T_{N}+F_{P}+F_{N}},$$

where $T_{P}$ represents the number of correctly classified rice grains, $T_{N}$ represents the number of accurately classified non-rice grains, and $F_{P}$ represents the number of misclassified rice grains, that is, the fraction that is not rice itself but is misclassified as rice. $F_{N}$ represents the number of misclassified non-rice, that is, the fraction that is rice itself but is misclassified as non-rice.

(10) $$MAE\left(y,\hat{y}\right)=\frac{1}{n}(\sum_{n}^{i=1}|y-\hat{y}\left|\right)$$

(11) $$MSE\left(y,\hat{y}\right)=\frac{1}{n}\left(\sum_{n}^{i=1}{\left(y_{i}-\hat{y}\right)}^{2}\right),$$

where *y* denotes the true value, $\hat{y}$ denotes the predicted value, and *N* denotes the total number of samples.

The comparison of experimental results includes a comparison of the counting results of rice in the original and improved algorithms for Categories A, B, and C, respectively, the 1000 g counting results of rice in Categories A, B, and C, and the 1000 g counting results of glutinous rice as a test model. The judging criteria were mean difference MAE, mean squared error MSE, and accuracy rate. After several experiments, it was found that, although the convergence of the improved algorithm was not fast, it was not more volatile and had better training results. The best model in training was used in the test set to obtain the mean MAE, and mean MSE. 1000 rice grains of three types of varieties and a new variety (glutinous rice) not in the training dataset were counted and tested simultaneously in this paper to analyze the results.

### 4.1. Performance of Class A Rice on the Original and Improved Algorithms

**Figure 6:**
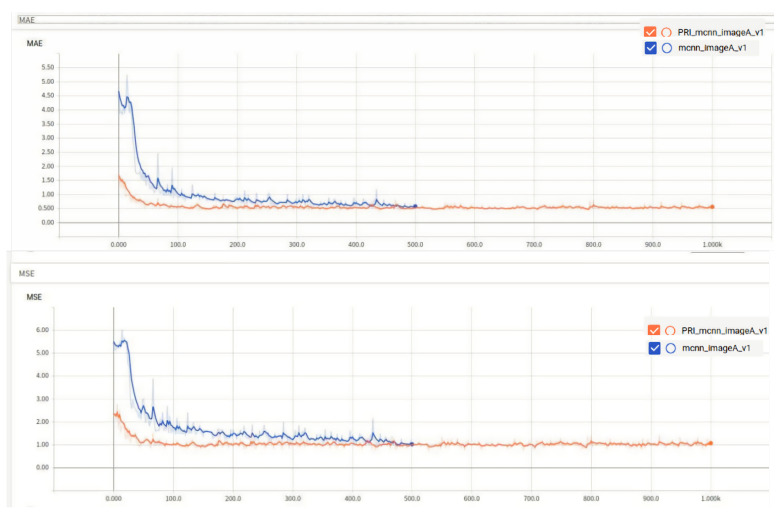
MAE and MSE of Class A rice on two algorithms, the blue curve represents the improved algorithm and the orange curve represents the original algorithm.

MAE and MSE denote the absolute mean error and mean squared error of the three types of the best model for A, B and C, in the training phase, respectively. The tests MAE and MSE represent the absolute mean error and mean squared error of the best model for A, B and C in the training phase, respectively, and smaller MAE and MSE are better. For example, the training MAE in the table is 0.4 in Class A rice, which means the absolute mean error in training for Class A rice is 0.4. The MAE and MSE of Class A rice on the two algorithms are shown in Figure 6.

**Figure 7:**
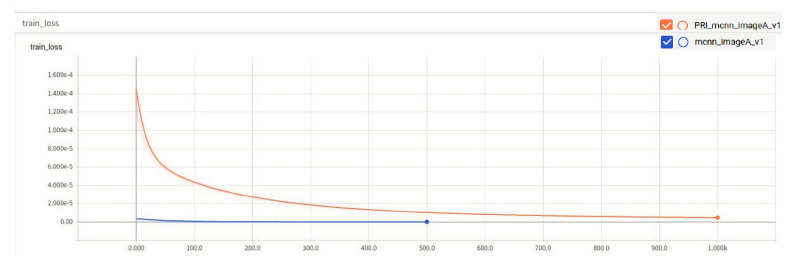
Training loss.

The comparison of the training errors of the two algorithms is shown in Figure 7, which indicates the error between the prediction results of the training set in the model and the real results.

The specific results of Class A rice on the original and improved algorithms are shown in Table 1.

**Figure 8:**
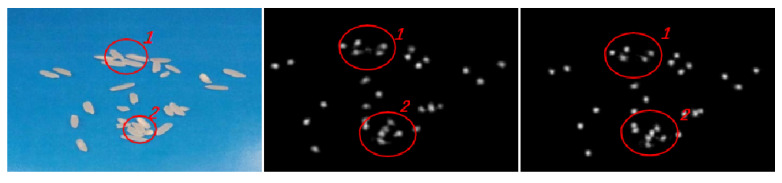
Original image and two kinds of algorithm detection results.

From the images, the performance of Class A rice in the original algorithm is initially better than the improved algorithm, but at around 470 epochs, the improved algorithm starts to perform comparably to the original algorithm. Although the MAE and MSE of the improved algorithm are only 0.1 lower than those of the original algorithm, as observed from the training results. The performance of the model of the improved algorithm on the test set is 1.49% better than that of the original algorithm, and the loss function of the improved algorithm is much smaller than that of the original algorithm, as seen from the train loss function, indicating that the improved algorithm is better than the original algorithm. The results of the two types of algorithms are shown in Figure 8.

For the generated density map, the density map of the improved algorithm is more accurate, especially as shown in Figure 1. When the original image of rice is connected tightly, the original algorithm cannot confirm whether it is the same rice or different rice and may generate some unnecessary density shadows, but this situation does not exist in the improved algorithm. In fact, for the improved algorithm, the whole density map generation is very clear, and there is almost no blurred density map generation. In this case, the density map is accurately generated to obtain more accurate coordinates, and the recognition rate of the improved algorithm is higher than the original algorithm.

### 4.2. Performance of Class B Rice on the Original and Improved Algorithms

**Figure 9:**
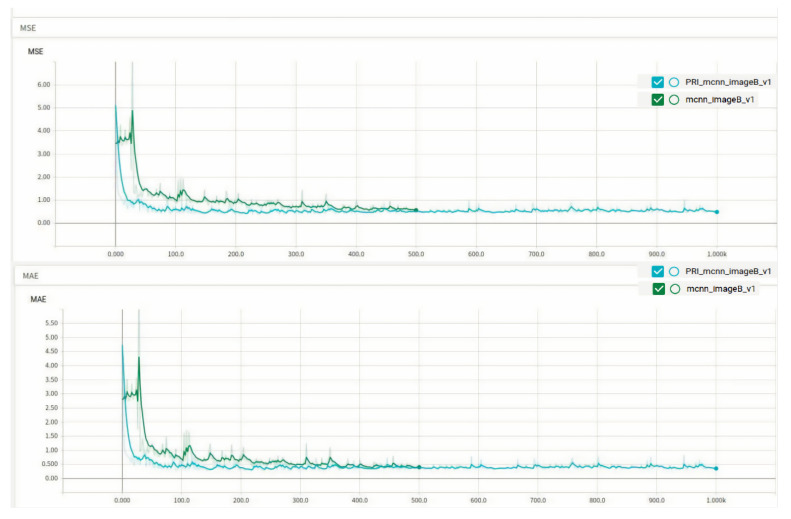
MSE of Class B rice on two algorithms.

The MSE and MAE of Class B rice on both algorithms are shown in Figure 9.

**Figure 10:**
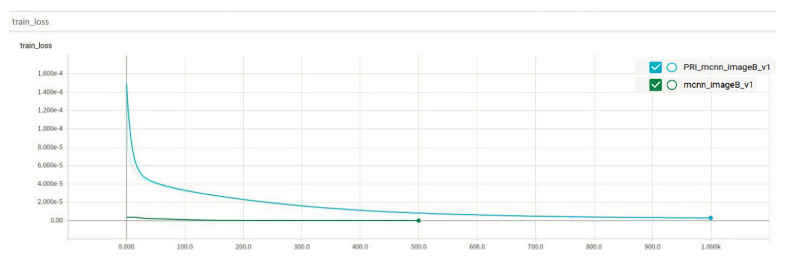
Train loss.

A comparison of the train loss of the two algorithms is shown in Figure 10.

The specific results of Class B rice on the original and improved algorithms are shown in Table 2.

**Figure 11:**
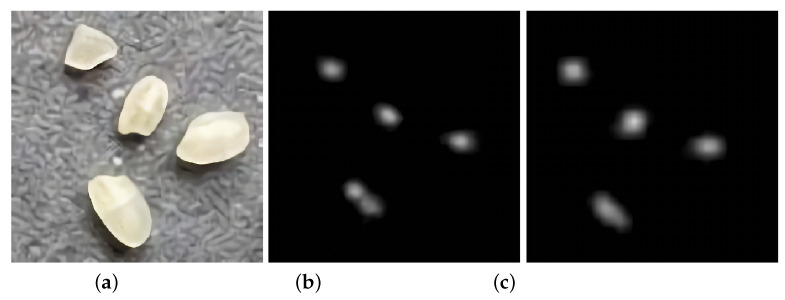
Class B rice labeling and comparison of density maps of two algorithms. (**a**) Original graph. (**b**) Density graph of the original algorithm. (**c**) Density graph of the improved algorithm.

Class B rice does not perform well in the improved algorithm at first, and the original algorithm converges quickly. After 480 epochs, the improved algorithm reaches the convergence level of the original algorithm and then remains in a mutually equal state. The performance of the improved algorithm on the test counts of Class B rice is greatly improved, with MAE reduced by 1.73 and MSE directly reduced by about half. To analyze the specific reasons, Figure 11 shows some of the original plots with the effect density plots of the two algorithms.

In this part of the original figure, there are four grains of rice. Observing the original figure, it is found that, because there is a more obvious and very regular split line in the middle of the B class rice, the color of the rice on both sides of the split line has a more obvious color difference. For this kind of adhesion, the original algorithm learns the feature that the rice with two characteristics is actually two grains of rice, so in the generation of the density map, the original algorithm generates two point coordinates for the same grain of rice. Therefore, in the density map generation, the original algorithm generates two point coordinates for the same grain of rice and considers it as “two grains of rice”. In the improved algorithm, because the amount of rice is estimated in advance, the overall amount of rice is found to be the same rice, and only one density coordinate is given in the generated density map.

Further experiments were conducted to investigate the counting effect of the original algorithm and the improved algorithm in relation to sticky rice. Images with a large amount of sticky rice were prepared for the experiments. Table 3 shows the counting performance of the original algorithm and the improved algorithm for this case of sticky rice. The table headings are the accuracy of counting sticky rice for the corresponding algorithm used, and the accuracy of counting rice for the image as a whole, respectively.

As can be seen from Table 3, the improved algorithm improves the accuracy of counting sticky rice by nearly 10% compared to the original algorithm. When the improved algorithm counts all rice, it can obtain an accuracy of more than 98%.

### 4.3. Performance of Class C Rice on the Original and Improved Algorithms

**Figure 12:**
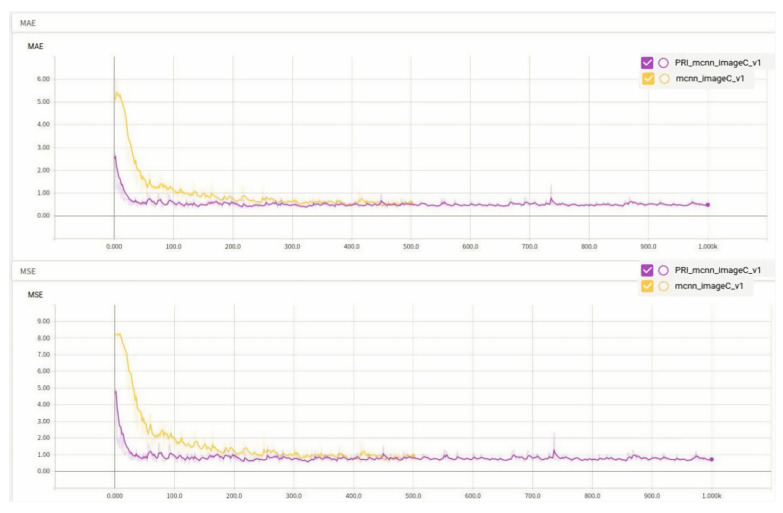
Comparison of MAE and MSE of Class C rice on two algorithms.

The MAE and MSE of Class C rice on both algorithms are shown in Figure 12.

**Figure 13:**
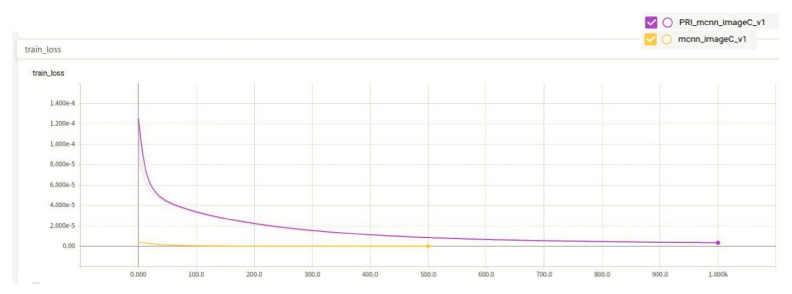
Training loss.

A comparison of the training loss of the two algorithms is shown in Figure 13.

The training and testing results of Class C rice on the original and improved algorithms are shown in Table 4.

**Figure 14:**
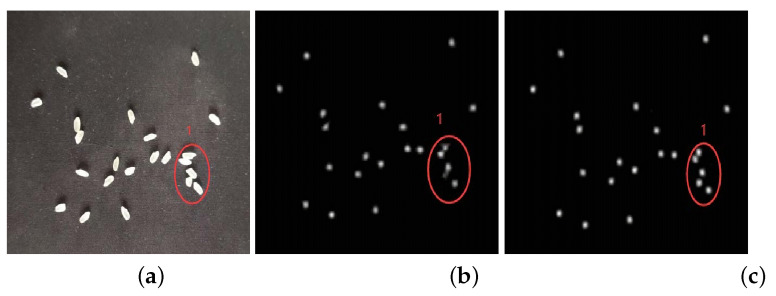
Comparison of density maps generated by Class C rice in the original algorithm and the improved algorithm. (**a**) Original marker map. (**b**) Original algorithm density detection map. (**c**) Improved algorithm density detection map.

In Class C rice, it is obvious that the accuracy of the improved algorithm is 3.14% higher than that of the original algorithm, the train loss of the improved algorithm is much smaller than that of the original algorithm, and the convergence speed is quite fast. The accuracy of the improved algorithm for Class C rice improved by 3.14% compared with the original algorithm, and Figure 14 shows the comparison of the density maps generated by the original and improved algorithms for a test image of Class C rice.

On the whole, the density map generated by the improved algorithm is clearer, and the improved algorithm is more “sure” of the exact location of the rice at 1 in the figure, while the original algorithm is unable to be “sure” of the rice with an incomplete and full shape, or the rice has overly small intervals when generating the density map. The density map generated is blurred or even shaded, which seriously affects the counting effect.

### 4.4. Rice Grains Test

#### 4.4.1. 1000 g Test on A, B, and C Rice

In this section, three types of rice, A, B and C, were prepared to test the feasibility of the counting method in this paper. Table 5 shows the test performance of 1000 g of the A, B, and C rice species in their best respective training models.

**Figure 15:**
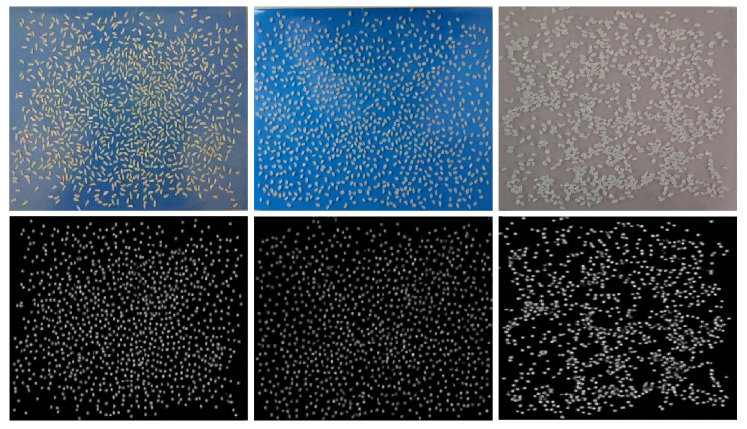
Generated density maps for each of the three types of improved rice algorithms. From left to right are the original input images of A, B, and C and the corresponding generated density maps.

Table 5 shows that the improved algorithm models for Species B and C have very good performance when the amount of rice increases. Figure 15 represents the original and improved algorithm’s generated density plots for each of the three rice types, A, B, and C, with 1000 g.

#### 4.4.2. Test Results of 1000 g of the New Variety (Glutinous Rice)

Table 6 shows the test results of the best model of Gnome in the three types of algorithms for A, B, and C.

For new varieties of glutinous rice that do not appear in the prepared dataset, the best-performing rice models of Class A in the test set performed average here. On the contrary, Table 4, Table 5 and Table 6 simultaneously show that the models of Rice Categories B and C had a very good counting ability when the amount of rice increased, and the generalization ability of these two types of models was stronger than that of Rice Category A.

### 4.5. Overall Analysis of Results

Based on the test set results and train loss, all three types of rice outperformed the original algorithm in the improved algorithm. Class A rice has the best performance on the test set, although its training model is not the best among the three categories in both the original and improved algorithms. Class A rice has a long bar shape, and the previous experiments showed that the counting algorithm based on the MCNN, the counting algorithm based on the MCNN and the density map, and its improvement algorithm were found to be the best for counting long-striped rice.

However, when the amount of rice increased, the counting ability of the Class A model was obviously insufficient. On the contrary, although the performance of the Class B and C models was poor on the test set, they had a very good counting effect on large-scale rice, and the generalization ability of the B and C models was also found to be very strong through the test on 1000 glutinous rice grains, which has excellent practical applicability.

## 5. Conclusions and Outlook

### Conclusions

As an important indicator for judging the quality of crop seeds, counting crop seeds is necessary to measure the thousand seed weight, so it is important to study the counting methods for crop seeds for measuring the thousand seed weight and crop quality. This paper firstly introduces the current research status and application fields of counting algorithms at home and abroad, and shows the importance of research on counting algorithms for rice in the context of current crop cultivation and needs. In this paper, two deep learning-based counting algorithms are used for rice, which are MCNN-based and density map-based counting algorithms, and an improved algorithm with advanced prior based on the original algorithm. After experiments, it was proved that both algorithms can count rice well. The effect of rice shape on the counting results was also investigated, and it was found that the long-striped indica rice have the best counting results. The two algorithms proposed in this paper have better migration. The main highlights of this paper are as follows:

1. The adaptive Gaussian kernel function used in generating the density map in this paper reduced the error when convolving rice of different shapes.
2. The improved MCNN network was based on the original network with the addition of advanced a priori training assistance, and the convergence and accuracy of the network were greatly improved compared to the original network.
3. The MCNN used was a combination of three independent and concurrent CNNs, which solved the problem that the filter extracts features of a certain size.
4. Since the last layer of the network structure of this paper uses a 1 × 1 size filter, the size of the input image was not required.
5. The dataset was collected and produced entirely by our researchers and is not reproducible.

In this paper, counting experiments were conducted on rice with good results. After the counting experiments for the three categories of rice, A, B, and C, some extension experiments were conducted, mainly the following:

1. The performance of the original algorithm and the improved algorithm was studied in relation to rice with too much sticking.
2. Cases of 1000 g of rice in Categories A, B, and C were tested, and the performance of the improved algorithm in cases where the amount of rice increases was verified.
3. Glutinous rice was used to study the generalization of the model in this paper.

In summary, in terms of innovation and experimentation, much effort was made in this study to demonstrate that the improved network has improved accuracy and in an improved migration ability. However, the algorithm proposed in this paper still has some shortcomings. Firstly, there is still much room for improving the accuracy of some species; secondly, the training time of the improved algorithm is quite long. At the same time, since the dataset was collected and produced by our team, there may be individual point labeling errors. The experimental object of this paper is rice, so cases of complete overlap are rare, but such a situation is not excluded from the realm of possibility. However, for seeds of the same species, characteristics are generally similar, so it is important to develop a model with a stronger generalization ability. Meanwhile, the counting algorithm in this paper can theoretically count other objects. It is equally important to extend the algorithm to count other objects. If the counting algorithm theory of this paper is transferred to other experimental objects, it is necessary to consider the use of a three-dimensional modeling approach to solve the problem of occlusion and overlap between objects. This is one of the most important directions for future development, and we will continue to conduct related research focusing on such problems.

## Figures and Tables

**Table 1:** Training and testing results of Class A rice on the original and improved algorithms.

Method	Original	Current Work
Train MAE	0.4	0.5
Train MSE	0.7	0.8
Test MAE	2.68	1.79
Test MSE	3.56	2.90
ACC	95.53%	97.02%

**Table 2:** Training and testing results of Class A rice on the original and improved algorithms.

Method	Original	Current Work
Train MAE	0.3	0.3
Train MSE	0.4	0.4
Test MAE	4.70	2.97
Test MSE	8.44	4.34
Accuracy	94.78%	96.70%

**Table 3:** Comparison of the counting performance of the original and improved algorithms on adhesion.

Method	Counting Accuracy of Conglutinated Rice	Counting Accuracy of All Rice
Original	76.76%	94.82%
Current Work	86.67%	98.30%

**Table 4:** Training and testing results of Class A rice on the original and improved algorithms.

Method	Original	Current Work
Train MAE	0.3	0.3
Train MSE	0.5	0.5
Test MAE	3.36	1.79
Test MSE	4.09	2.30
Accuracy	93.28%	96.42%

**Table 5:** Accuracy of the 1000 g test results for Categories A, B, and C.

Classification of Rice	Original	Current Work
A	94.15%	97.52%
B	93.50%	99.52%
C	92.01%	99.13%

**Table 6:** Performance of 1000 g of glutinous rice.

Classification of Rice	Original	Current Work
Model A	93.08%	96.21%
Model B	93.01%	97.67%
Model C	93.08%	98.74%

## Data Availability

Not applicable.
